# Virus-like particle: a nano-platform that delivers cancer antigens to elicit an anti-tumor immune response

**DOI:** 10.3389/fimmu.2024.1504124

**Published:** 2025-01-07

**Authors:** Weisen Ning, Sheng Yan, Yongyao Song, Hanning Xu, Jinling Zhang, Xiaomei Wang

**Affiliations:** ^1^ School of Medicine, Wuhan University of Science and Technology, Wuhan, Hubei, China; ^2^ Department of Oncology, Wuhan Fourth Hospital, Wuhan Orthopedic Hospital, Wuhan, Hubei, China

**Keywords:** virus-like particles, tumor immunotherapy, tumor vaccine, tumor antigens, nanoplatform

## Abstract

Virus-like particles (VLPs), as a unique form of nanocarrier, predominantly encompass hollow protein shells that exhibit analogous morphology and structure to naturally occurring viruses, yet devoid of genetic material. VLPs are considered safe, easily modifiable, and stable, making them suitable for preparation in various expression systems. They serve as precise biological instruments with broad applications in the field of medical biology. Leveraging their unique structural attributes and facile modification capabilities, VLPs can serve as an effective platform for the delivery of tumor antigens, thereby stimulating the immune system and facilitating the eradication of tumor cells.

## 1 Introduction

Vaccines are one of the most successful and cost-effective interventions available to control and prevent infectious diseases (1). The conventional vaccine approach chiefly targets viruses associated with corresponding diseases, typically utilizing attenuated or inactivated viruses. Upon entry into the host organism, they can stimulate efficacious T cell and B cell responses, potentially leading to long-term immunity. With the evolution of medical technology, these vaccines are progressively being utilized in the management of chronic diseases and malignancies. Currently, FDA-approved cancer vaccines primarily aim to prevent malignancies caused by infections with the hepatitis B virus (HBV) and human papillomavirus (HPV) (2), such as Dynavax, Cervarix and Gardasil 9. These preventive vaccines are specifically designed to inhibit infection by cancer-associated viruses. In fact, the development of therapeutic cancer vaccines is considerably more complex than that of preventive vaccines, as tumors often evade immune surveillance during their progression. Therefore, therapeutic cancer vaccines must possess the capability to induce a robust T-cell response against tumor cells while eliciting high and sustained antibody titers that can suppress cancer cells. A major challenge in this field lies in comprehending the diversity of tumor antigens and developing innovative technologies for activating innate immune responses and enhancing antigen delivery systems (3, 4). Therefore, it is especially pivotal to discover an antigen delivery platform that can amplify immunogenicity without jeopardizing safety, tolerability, and efficacy in the context of existing tumor antigens. Nanocarriers have been widely studied in the field of drug delivery, and are primarily used for the delivering chemotherapy (5) or nucleic acid drugs (6). A major challenge in drug delivery is the immunogenicity of the vector itself, which may lead to premature degradation within the body and reduce therapeutic efficiency (7). However, when nanocarriers deliver tumor antigens, their inherent immunogenicity can be exploited to effectively activate the immune system and achieve anti-tumor effects. In recent years, various strategies based on nanocarriers have been explored for antigen delivery including liposomal nanoparticles (LNPs) (8), inorganic nanoparticles (9), polymeric nanoparticles (PNPs) and VLPs (10).

LNPs are mainly spherical entities with a phospholipid bilayer shell and an aqueous core. To enhance their immunogenicity as vaccine carriers, the particle surface must be modified with ligands, antigens, or other lipids (11, 12). However, liposomes lack tissue selectivity and surface modifications can increase complexity and cost. Additionally, cationic lipids can exhibit cytotoxic effects on cells at high concentrations. Inorganic nanoparticles, such as gold and silica, persist for longer durations in tissues thereby potentially enhancing antigen presentation. Gold nanoparticles are often used for vaccine delivery but require electroporation for intracellular delivery, which can cause cell death and limit clinical use (13, 14). Commonly studied polymer-based nanoparticles include poly (D, L-lactide-glycolide copolymer) (PLG) and polylactide (PLA) (15, 16). Which are biodegradable and biocompatible. They are clinically approved for various implants (16, 17) or sutures are investigated for delivering vaccine antigens (18). Antigens can be embedded or adsorbed onto the particles, serving as a reservoir for gradual release of the envelope antigen (19). Additionally, PNPs can safeguard the delivered envelope antigen from oral degradation and enhance M cell uptake in nasal-associated lymphoid tissues (NALT) when administered intranasally (20, 21). However, scaling up PNPs production is challenging due to their nanoprecipitation process occurring under highly dilute conditions with very low solids content (22). Furthermore, limited characterization methods and intracellular assays hinder our understanding of PNPs behavior within intricate human biological systems.

As protein-based nanoparticles, VLPs exert protective effects by using their own mechanisms and structural principles to trigger the immune system, for example, by providing materials that mimic specific viral characteristics to stimulate immune responses. However, unlike actual viruses, VLPs typically consist of viral structural proteins but lack the infectious protein coats. These structural proteins play a critical role in VLPs and serve as the basis for their self-assembly into intricate structures (23). If the modification such as tumor epitopes is introduced in the production process, the body can produce anti-tumor immune response. VLPs are highly organized nanostructures that typically consist of a shell made up of one or more identical protein subunits arranged in specific spatial conformations, such as helices, icosahedra, spheres, or complex shapes (24). They can be produced in various expression systems, including mammalian cell lines (25), yeast (26), plants (27), insects (28) and prokaryotic cells (29). Structural proteins of various viruses can be used to produce VLPs, including bacteriophage (30), adeno-associated virus (AAV) (31), HBV (32), hepatitis C virus (HCV) (33), human immunodeficiency virus (HIV) (34), HPV (35), hepatitis E virus (HEV) and others (36). As mentioned above, VLPs are often able to be applied to multiple uses due to their unique structural properties. The hollow architecture generated by viral structural proteins facilitates their function as carriers for diverse payloads, encompassing genes, peptides, proteins, and small molecules. Furthermore, VLPs exhibit morphological diversity and frequently mimic the appearance of viruses. The pathogen-like associated structural pattern (PASP) augments their internalization by antigen-presenting cells (APCs) (37, 38), making it superior to other nanoparticle for antigen delivery. VLPs are divided into two main categories: envelope VLPs and non-envelope VLPs (39). Enveloped VLPs possess a lipid envelope derived from the host cell membrane. The presence of this envelope renders these particles morphologically and functionally more akin to authentic viruses, enabling them to participate in processes such as membrane fusion and cellular invasion. In contrast, non-envelope VLPs, which are composed only of the coat proteins of the virus, are usually structurally stable and not susceptible to environmental factors. Because the envelope of VLPs may contain a variety of antigens and induce a more complex immune response, so that non- envelope VLPs are usually used in the preparation of vaccines for specific antigens.

**Figure 1:**
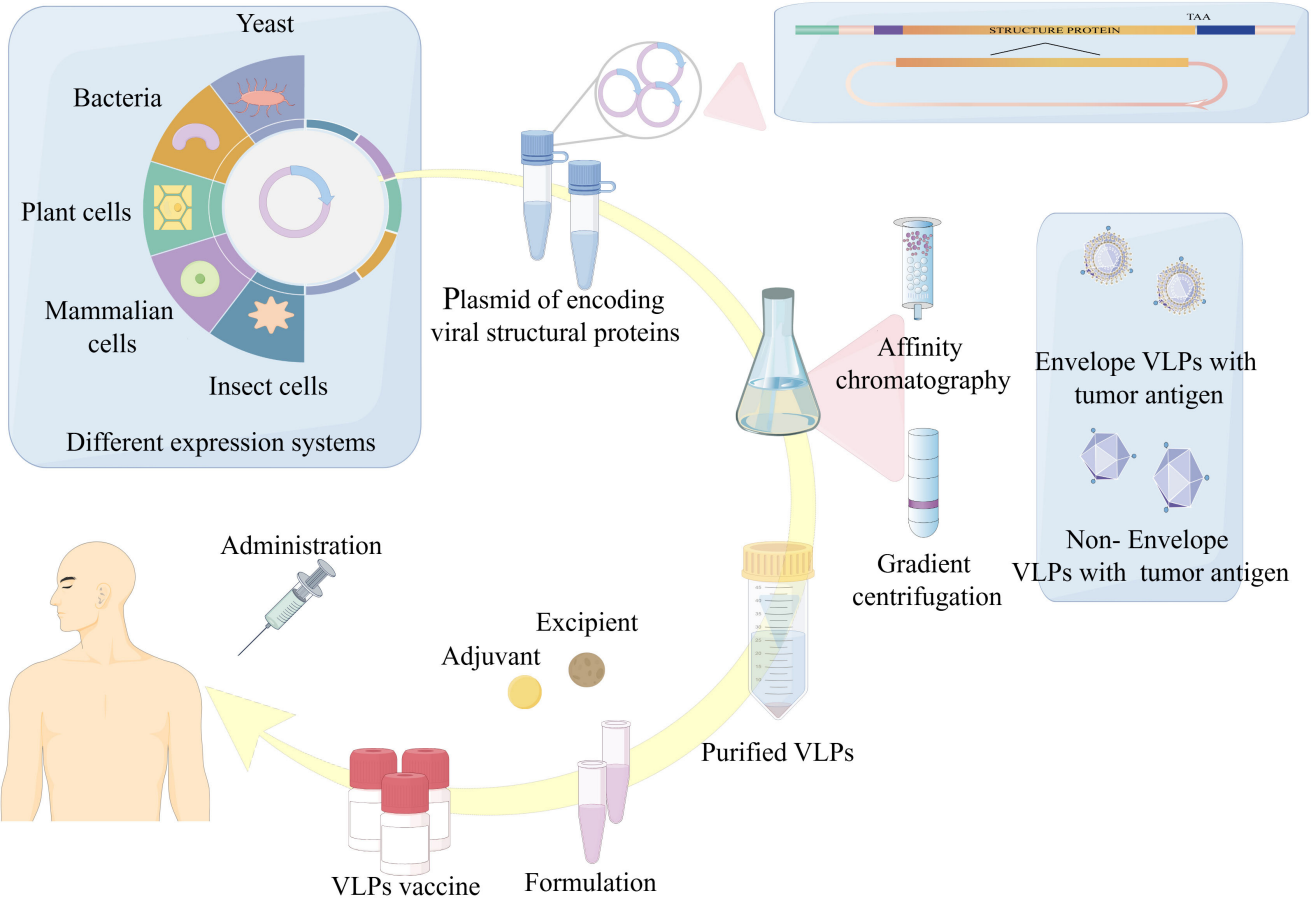
Overview of VLPs-based vaccine expression, purification and formulation. The production of tumor vaccines based on VLPs is mainly through: (1) Cloning of the viral structural genes of interest and expression of viral proteins with self-assembling ability in a suitable expression platform. (2) Purification of VLPs. (3) Adjuvant and additional ingredients are added to the vaccine formulation to finally achieve a safe, efficient and effective product for vaccination.

The preparation of VLPs as tumor antigen delivery agents generally involves three sequential steps: (A) production and expression, (B) purification, and (C) customization. Initially, the viral structural genes are cloned, followed by expression of self-assembling viral structural proteins within a suitable expression system. Thereafter, to achieve VLPs with high purity and integrity, ion-exchange chromatography or ultracentrifugation is commonly employed for further purification. Finally, aseptic filtration and formulation occur, during which adjuvants and other components are commonly integrated into the formulation to ensure a product that is safe, efficacious, and effective. Modifications such as incorporation of tumor antigens are usually introduced during the initial cloning step (**Figure 1**). In this review article, we briefly present the main developments in VLPs, as tumor antigen delivery platform in the prevention and therapeutic of cancers, as well as their future prospects, are discussed.

## 2 Cancer therapeutic antigens for VLPs-based tumor antigen delivery platform

One of the crucial issues that necessitate addressing for a VLPs-based tumor antigen delivery platform is the selection of the suitable antigen for efficient delivery *in vivo*. The loading of tumor antigens endows VLPs with the capability to stimulate tumor antigen-specific immune responses, conferring on the immune system an “aiming ability” allowing the immune system to precisely targeting cancer cells bearing tumor antigens. Suitable antigens enhance the precision of VLPs targeting and minimizes or prevents damage to healthy cells. Tumor antigens are typically categorized into two classifications according to their specificity: tumor-associated antigens (TAA) and tumor-specific antigens (TSA). The former represents an antigen that is minimally expressed in normal cells yet highly expressed in tumor cells, whereas the latter is uniquely expressed in tumor cells and is lacking from other normal cells. As research advanced, the scientists delineated tumor antigens into distinct categories: cancer-testis antigen (CTA), neoantigen, oncoantigen and so on.

CTA represents an ideal and promising target for cancer therapy. This multifunctional protein group exhibits a specific expression pattern in normal embryonic tissues, adult cells and various types of cancer cells. CTA plays a crucial role in regulating fundamental cellular processes such as development, stem cell differentiation, and tumor formation. However, its specific biological and cellular functions remain incompletely understood (40). Among the over 60 genes encoding *cta*, the most widely studied include the melanoma-associated antigen family, sarcoma antigen 1 and testicular cancer antigen 1 (3). Neoantigens are proteins uniquely present in tumor cells generated from mutations in the tumor cells DNA (41–43). These mutations may include single nucleotide variations, base insertions or deletions, and gene fusions. Neoantigens are recognized as non-self-entities, offering distinct advantages over other categories of tumor antigens. Since neoantigens result from mutations in the DNA of tumor cells, and these mutations are unique to individual tumors, neoantigen-based vaccines must be formulated independently for each patient’s specific tumor (44). Oncoantigens are typically delineated as persistent tumor antigens that assume a causal role in tumor progression and do not circumvent immune recognition (45). The expression levels of certain biomarkers, such as EGFR, HER2, mucin MUC1 and insulin-like growth factor-1 receptor (IGF1R), are reduced in healthy cells but elevated in tumor cells. Presently delineated cancer antigens are classified into three categories. Class I antigens are localized on the plasma membrane of tumor cells, implying that they are surface proteins or molecules that can be readily recognized by the immune system. Class II antigens are not directly expressed by neoplastic cells, but are present within the tumor microenvironment, including cells or molecules influenced by the cancer. Class III antigens are intracellular not directly exposed to the immune system; however, their fragments can be recognized by immune cells through cellular processing and presentation mechanisms (46).

Neoantigens represent the most promising targets among tumor antigens, and the most extensively researched neoantigens for vaccines and immunotherapy are clonal neoantigens of KRAS, BRAF, and PIK3CA driver genes. Cancer vaccines incorporating neoantigens exhibit greater patient specificity. This highly personalized therapy relies on the identification of mutations, the prediction of potential neoepitopes, and the design and manufacture of vaccines. The rapid and cost-effective detection of tumor-specific mutations in individual patients through next-generation sequencing, coupled with the development of algorithms to predict MHC molecule binding epitopes, has enabled the identification of potential immunogenic neoepitopes. The modifiability of VLPs facilitates their artificial alteration to incorporate tumor antigens via related technologies, thereby achieving recognition by immune cells upon systemic entry, stimulating the immune system to specifically eliminate tumor cells. VLPs-based antigen delivery platforms are generally considered safer and more stable than traditional vaccines due to their structural similarity to viruses but lack of genetic material (47). Additionally, their small size (25-500 nm) facilitates entry into the lymphatic system (48, 49). Owing to these important features, when VLPs are genetically or chemically engineered to display repetitive and densely packed tumor epitopes, these epitopes induce robust antitumor immune responses. This undoubtedly represents a formidable vaccine strategy.

## 3 Bioengineering strategies for VLPs-based antigen delivery platforms

**Figure 2:**
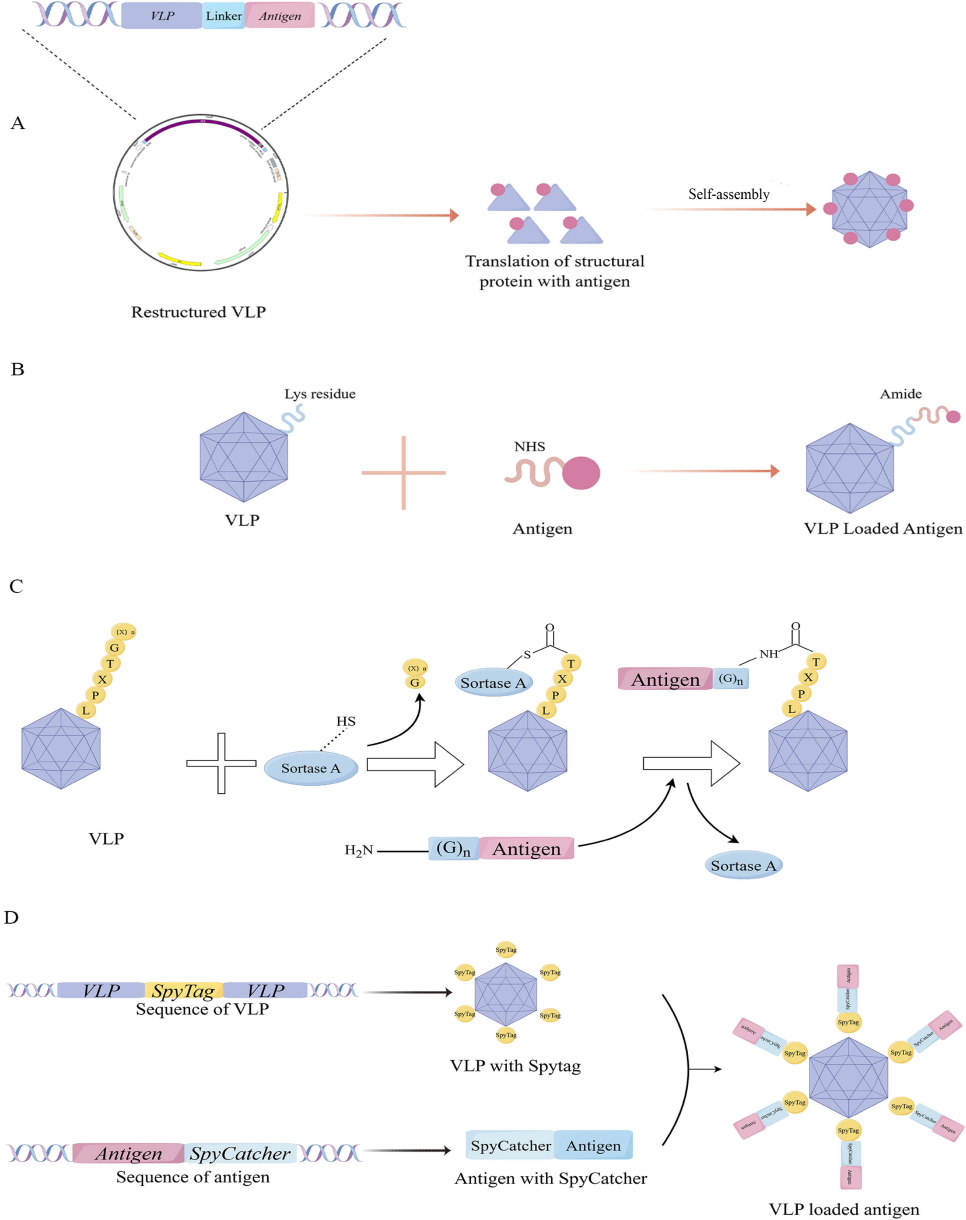
Conjugation method of VLPs with tumor antigens. **(A)** Gene fusion to link antigens; **(B)** Amino acid conjugation. **(C)** Mechanism of antigen ligation using the Sortase technology. **(D)** Mechanism of antigen ligation using the Spy-Catcher technology.

To fully exploit the immunogenic potential of particle platform technology, it is necessary to select an appropriate conjugation strategy that optimally presents tumor antigens of interest on VLPs. Currently, various strategies are used in research, but the specific approach should be tailored to the VLPs’ structural characteristics (**Figure 2**). The advantages and disadvantages of some strategies are summarized in **Table 1**.

**Table 1:** Advantages and disadvantages of some biological linking strategies.

Strategy	Advantage	Disadvantage
Gene recombination fusion	Easier preparation Low cost and easy to express in large quantities	May result in misfolding Lack of universality
Amino acid conjugation	Procedure is simple and does not lead to protein misfolding	The chemical reaction is uncontrollable
Sortase A Technology	The specificity was high and the reaction was controllable	There are certain requirements for the substrate sequence that may limit its application to some proteins
Spycatcher technology	The ability to bind antigens of high molecular weight	More complex operation
Cu-free click chemistry	Non-toxic, safe, and able to display a variety of antigens	Will increase production costs and affect the scalability of vaccine production
His-Tag/Ni-NTA Affinity	The reaction conditions were mild and conducive to maintaining the structure of the protein	Higher cost

### 3.1 Gene recombination fusion for VLPs-based antigen delivery

By utilizing genetic engineering, exogenous peptides can be incorporated into VLPs through sequence insertion, allowing concurrent peptide display during VLPs expression — a method that is widely utilized at present (50). While this approach facilitates the preparation of VLPs carrying tumor antigens, the direct insertion of foreign sequences often results in the misfolding of recombinant proteins, potentially affecting VLPs assembly and their other biological functions. Therefore, it is crucial to identify a viral structural protein with a stable structure and an appropriate method for peptide display. One extensively studied instance is VLPs derived from HBV core antigen (HBcAg). HBcAg-VLPs self-assembled from 180 or 240 core protein subunits into a regular icosahedral particle structure. Billaud et al. significantly increased the success rate of chimeric HBcAg-VLPs with multiple insertion sites, compensatory mutations, and efficient screening (51). Although some general rules can be summarized, these findings remain contingent upon the expertise of the investigators and subject to some chance. In another study of phage MS2 VLPs, peptides are typically inserted within the surface ring (AB loop), causing structural instability of the wild-type protein shell. Jerri C Caldeira’s team discovered that while the coat protein of RNA phage MS2 typically does not tolerate insertions in its AB loop, an engineered single-chain dimer readily accepts such modifications as long as they are confined to one of its two halves. The study found that genetically engineered single-stranded dimer protein shells enhance thermodynamic stability through covalent linkage between two non-covalent subunits (52). It is evident that the technique of linking antigens through gene fusion frequently relies on an in-depth understanding of the structural characteristics of the VLPs used in the experiment, and this method lacks universality.

### 3.2 Amino acid conjugation for VLPs-based antigen delivery

Another method, known as amino acid conjugation, is also widely used by researchers. This technique mainly relies on the chemical conjugation of specific amino acid residues exposed on the surface of VLPs to form covalent bonds with antigens possessing complementary reactive groups to achieve the purpose of linking. For instance, the lysine residue of VLPs can be conjugated to the target antigen through its reaction with N-hydroxysuccinimidyl ester (NHS), forming a stable amide bond. Alternatively, the sulfhydryl group of the cysteine side chain binds can react with maleimide to create an irreversible and stable thioether bond, effectively linking the protein of VLPs to the target antigen. While cysteines are rare on the surface of general proteins, they can be artificially introduced via genetic engineering techniques, such as direct insertion or site-directed mutagenesis. Notably, classical amino acid-conjugated VLPs-based vaccines, including bacteriophage Qβ and tobacco mosaic virus, are currently undergoing clinical trials (53–55). These methods are characterized by their simplicity and cost-effectiveness. However, the inherent lack of control over the chemical reactions can lead to issues, particularly when the introduced reactive non-natural amino acids interfere with the formation of pre-existing disulfide bonds within the antigen.

### 3.3 Other technologies for VLPs-based antigen delivery

To achieve greater control over the ligation reaction, several novel approaches have been reported (**Figure 2**). Sortase is a highly conserved and widely distributed class of membrane proteins and virulence factors present in gram-positive bacteria, such as *Staphylococcus aureus*, which harbors the sortase A. They play a crucial role in facilitating bacterial invasion into host cells and forming bacterial biofilms (56, 57). In *Staphylococcus aureus*, sortase A promotes the localization of various bacterial proteins to the bacterial surface through transpeptideization. Specifically, it can recognize the C-terminal peptide sequence LPXTG of the substrate protein and form an amide bond with the N-terminal glycine residue, making it an effective tool for VLPs modification (57, 58).

Spycatcher and Spytag, serving as the principal effector molecules in the SpyCatcher/SpyTag covalent fusion technology, are derived from the CnaB2 domain in streptococcal fibronectin FbaB. Spycatcher, composed of 113 amino acids with active Lys31, binds stably with Spytag, which contains 13 amino acids with active Asp117, forming intermolecular isopeptide bonds (59). Fusion of Spytag and Spycatcher to the N or C termini of proteins and VLPs enables molecular ligation. These distinctive characteristics have paved the way for innovation strategies in protein engineering and opened new avenues for biological research and therapeutic applications. For example, Ming-Hao Yang’s group developed a Bamboo Mosaic virus (BaMV)-based binary epitope presentation CVP platform using sorting enzyme A (SrtA)-mediated ligation technology to display the recombinant envelope domain III (rEDIII) of Japanese encephalitis virus (JEV) (58). And Tiong Kit Tan’s group used SpyCatcher technology to display the coronavirus spike glycoprotein receptor binding domain (RBD) on a synthetic VLPs platform SpyCatcher003-mi3 (60).

Additionally, Cu-free click chemistry has been gradually applied to construct recombinant VLPs as a safe and effective method (61–63). Dibenzocyclooctyne (DBCO), the most reactive cycloalkyne, is commonly used for alkyne-azide cycloaddition reactions. DBCO binds to peptides via amino, sulfhydryl, or carboxyl groups. This azide-based click chemistry is generally safer than earlier copper-catalyzed methods because it does not require copper. While these methods ensure controlled reactions and stable VLPs folding, they may increase production costs or limit VLPs-based vaccine scalability.

Another approach involves non-covalent modification, such as the method used by Hytonen et al., who utilized the interaction between a His-tag and nickel-loaded tris-subazoyl triacetic acid (trisNTA) to modify norovirus VLPs (64). The binding affinity of His-tag to Ni-NTA is moderate, and this interaction weakens at lower pH due to the increased protonation of histidine residues. Consequently, the VLP assembly may be susceptible to rearrangement during vaccine storage or in the low pH environment following injection.

## 4 Mechanisms of antitumor immune responses induced by VLPs-based tumor antigen delivery platform

**Figure 3:**
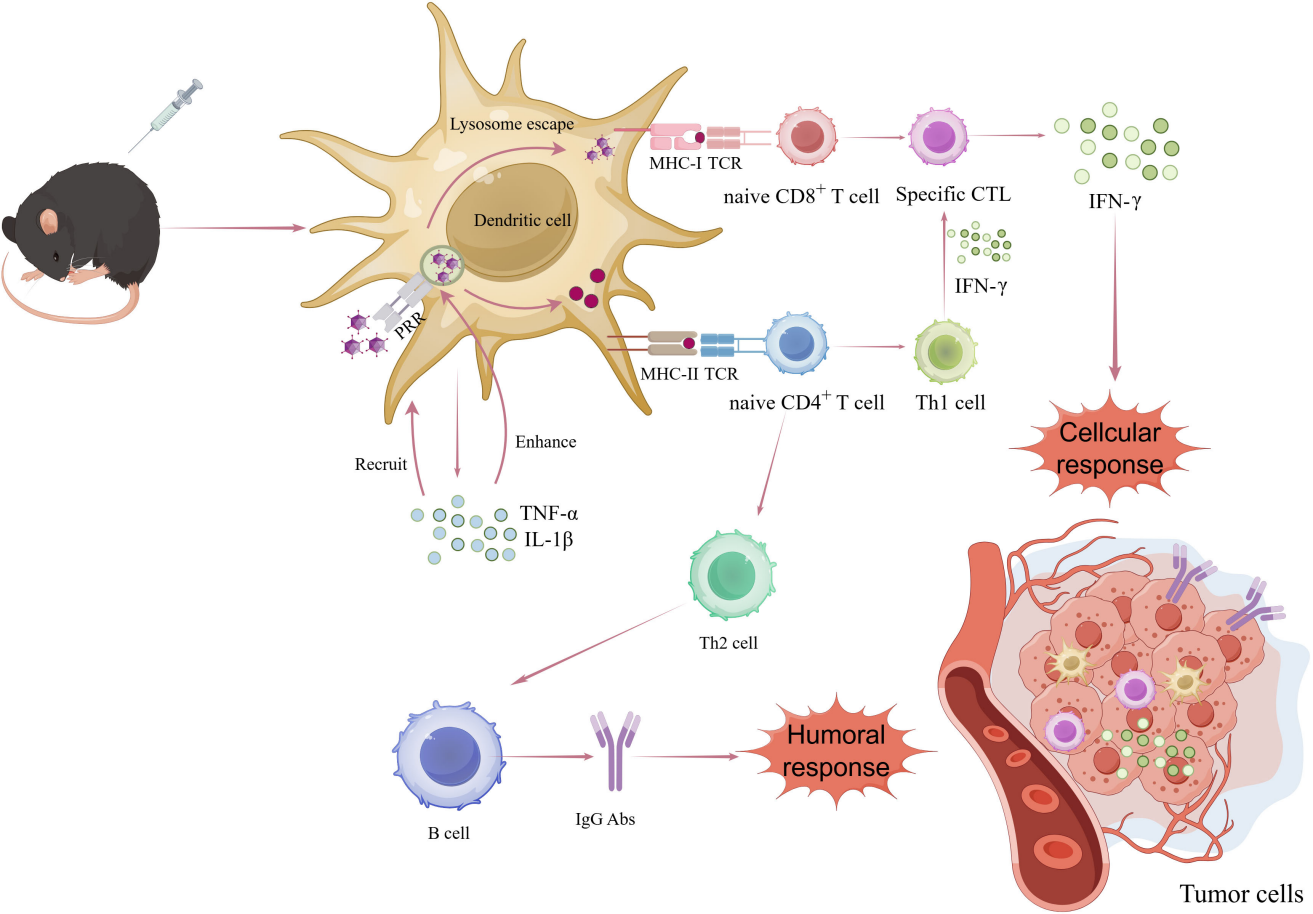
Mechanisms by which VLPs-based antigen delivery systems activate antitumor immune responses. After being phagocytosed and processed by dc through the PRR-mediated endocytic pathway, VLPs are presented to MHC-I and MHC-II for recognition by CD8^+^ and CD4^+^ T cells. CD4^+^ T cells differentiate into TH2 and TH1 cells that are involved in inflammatory response and in sustaining the activity of CD8^+^ T cells (cytotoxic T cells), respectively. CD8^+^ T cells exert cytotoxic activity on tumor cells.

The induction of a robust adaptive immune response is essential for an effective anti-tumor immune response, intricately associated with antigen presentation by DCs. DCs, among the most important APCs, are critical in initiating adaptive immune responses. They can internalize particles as small as 100-500 nm (65, 66). VLPs, as a type of nano-platform, can effectively present key epitopes to DCs. Among various DCs subsets, conventional type 1 DCs (cDC1s) are particularly vital in anti-tumor immune responses. Following immunization with VLPs, DCs interact with them via pattern recognition receptors (PRRs), such as toll-like receptors and C-type lectin receptors. DCs internalize VLPs through endocytosis and transport them to secondary lymphoid tissues. Upon uptake and recognition of VLPs, DCs mature, leading to the production of TNF-α and IL-1β (67). These proinflammatory factors recruit more APCs and enhance lysosomal proteolysis in DCs. Most VLPs carrying antigens enter the body and are transported to the draining lymph nodes, where they are internalized by DCs through receptor-mediated endocytosis. Subsequently, they are guided to early phagosomes or endosomes, releasing antigens for processing and presentation via the MHC-I and/or MHC-II pathways. Exogenous antigens are typically presented by MHC-II molecules, while endogenous antigens are presented by MHC-I molecules (68). At the same time, lymphocyte costimulatory molecules (e.g. CD80, CD86) appear on DCs surfaces to activate B and T cells. The activation and proliferation of B cells, leading to humoral immunity, are facilitated by MHC-II peptide complexes and costimulatory molecules interacting with CD4^+^ T helper cells. However, VLPs can also directly stimulate humoral immunity by specifically binding to naive B2 cells and their antigen receptors, thereby inducing upregulation of CD69 and CD86 without relying on helper T cells (69, 70). Although humoral immunity plays an important role in anti-tumor responses by producing neutralizing antibodies, it appears to be just one of the immune pathways involved. It was demonstrated that the recombinant bacteriophage P22-VLPs vaccines carrying the model antigen OVA can be cross-presented by MHC-I, thus activating CD8^+^ T cells (71). The subsequent activation of CD8^+^ T cells into cytotoxic T lymphocytes (CTLs). *In vivo*, the anti-tumor immune response is mainly mediated by CTLs, with the primary objective of delivering tumor antigens being to activate these cells to stimulate the immune system (72). To validate the efficacy of VLP-OVAT in stimulating activated DCs for antigen processing and presentation to CD8^+^ T cells, Wenjing Li et al. conducted CFSE dilution experiments by using transgenic OT-1 T cells. After intravenous injecting CFSE-labeled OT-1 CD8^+^ T cells into naive mice, immunization was administered in the groin area on the following day. Subsequently, flow cytometry analysis showed a significant increase in OT-1 T cell proliferation and a reduction in the CFSE signal specifically in the P22-VLP-OVAT group, while no substantial changes were observed in the P22-VLP-WT or free OVAT peptide control groups. These findings are consistent with prior *in vitro* experiments, indicating efficient processing and cross-presentation of P22-VLP-OVAT by DCs, leading to the successful expansion of specific CD8^+^ T-cells (71). Additionally, CD4^+^ T cells play a crucial role in activating CD8^+^ T cells and enhancing tumor immunity, particularly through their differentiation into the Th1 subtype, which maintains CD8^+^ T cell activity. Studies in mouse models have elucidated the effects of CD4^+^ T cells on cytotoxicity (73), migratory and invasive capacity of CTLs, and downregulation of co-inhibitory receptors. Furthermore, effector and memory CTLs differentiation programs activated by helper signals may provide new antibody targets to promote the CTLs response to cancer, as well as diagnostic markers to assess vaccine efficacy (74) (**Figure 3**).

## 5 Preclinical studies of VLPs-based tumor antigen delivery platform

Tumor cells can evade the recognition of the immune system through various means, thereby achieving immune tolerance. The ultimate objective of cancer vaccines utilizing VLPs is to effectively disrupt this state of tolerance. VLPs serve as an effective antigen delivery platform with robust immunogenicity, capable of delivering large quantities of specified tumor antigens, including short peptides that may be rapidly cleared. This facilitates the presentation of cancer antigens to activate tumor antigen-specific immune responses and ultimately enables the immune system to eliminate malignant cells. The current cancer treatment or prevention strategy is still undergoing clinical trials, and it has also been extensively researched in the context of breast cancer, melanoma, and cervical cancer. In this section, we will primarily focus on presenting preclinical research findings related to these three types of cancer, while information regarding other types of cancer will be provided in **Table 2**.

**Table 2:** Some preclinical studies of VLPs-based vaccine.

Cancer Type	VLP Platform	Tumor Antigen(s)	Antigen carrying technology	Expression System	Adjuvant	References
Breast cancer	AP205 phage	HER2	Spy/Catcher	*E. coli*	None	(75)
	MS2 phage	xCT	Genetic engineering	*E. coli*	None	(76)
	HBcAg	IL-33	Genetic engineering	*E. coli*	None	(29)
	AP205 phage	HER2	Spy/Catcher	Fruit flyS2	Montanide ISA 51	(77)
Melanoma	HBcAg	Mage-3 (168-176)	Genetic engineering	*E. coli*	None	(78)
	RHDV	Gp100	Genetic engineering	Baculovirus	Mannosylation of the VLPs	(79)
	HBcAg	OVA257-264/gp100	Genetic engineering	*E. coli*	None	(80)
	CuMVT	p33	Cu-free chemical click	*E. coli*	Microcrystalline tyrosine	(81)
	Qβ phage	Germline and mutated epitopes	Cu-free chemical click	*E. coli*	B-type CpGs	(62)
	HAdV-3	OVA257-264 OVA 323-339	Spy/Catcher	Baculovirus	ODN 2395/MPLA/poly(I:C)	(82)
Cervical cancer	HPV	E6/E7	Genetic engineering	Plant cell	None	(83)
	HBcAg	E7	Genetic engineering	*E. coli*	None	(84)
Lung cancer	HIV Gag	Trop-2	Genetic engineering	Baculovirus	CD40L	(85)
	RHDV	gp33	Genetic engineering/Chemical coupling	Baculovirus	None	(86, 87)
Colorectal cancer	RHDV VP60	Topoisomerase IIα and survivin	Genetic engineering	Baculovirus	CpGs	(88)

### 5.1 Breast cancer

Breast cancer, with a high mortality rate, ranks as the most prevalent malignancy among women (89). Current research suggests that human epidermal growth factor receptor-2 (HER2), is overexpressed in around 20-30% of breast cancer cases, making it a highly promising TAA and an ideal target for therapeutic tumor vaccines (90, 91). Previously, monoclonal antibodies like Trastuzumab (Herceptin^®^) (92) and Pertuzumab (Perjeta^®^) (93), targeting the extracellular domain (ECD) of HER2, have effectively improved overall survival rates in patients with HER2-positive breast cancer (94). The use of monoclonal antibodies in therapy is expensive and requires continuous administration. However, prolonged exposure to high doses may lead to allergic side effects and resistance development (95). Arianna Palladini’s group effectively exploited the ability of SpyTag to interact with Spycatcher to form isopeptide bonds by using SpyCatcher’s antigen display technology, Spycatherer-her2 ECD fusion antigen was adsorbed on the surface of AP205 phage-derived VLPs. This innovative vaccine enables each VLPs to carry an average of 360 units of HER2 ECD epitopes. This vaccine effectively elicited an anti-HER2 immune response, resulting in the inhibition of growth in breast cancer cells expressing human HER2 in a murine breast cancer model. The findings underscore the efficacy of multivalent display of TAA on VLPs as a promising strategy to overcome B cell tolerance, thereby offering substantial insights (75).

Another potential therapeutic target for breast cancer is xCT, a cystine-glutamate antitransporter that is overexpressed in various human tumors but absent in healthy breast tissue. Moreover, xCT plays a role in maintaining breast cancer stem cells (CSCs) by increasing intracellular GSH concentrations. Inhibiting p38/mitogen-activated protein kinase activation reduces ROS levels, preventing CSCs apoptosis and promoting tumor progression (96). Currently, the vaccine named AX09 is progressing to the clinical development stage. The researchers genetically engineered the phage MS2 coat protein by incorporating the ECD3 peptide of human xCT protein into its ab ring. After immunizing BALB/c mice, the binding affinity of AX09 to human ECD3 peptide and its vaccine-induced humoral response were evaluated. It was observed that AX09 elicited robust levels of anti-XCT IgG1 and IgG2a, which effectively suppressed xCT function and attenuated tumor metastasis in mouse (76). The potentiated inhibition of xCT function holds the potential to enhance the chemosensitivity of breast cancer cells, thereby proposing a promising strategy for combination treatment (97).

### 5.2 Melanoma

Melanoma, a type of skin cancer originating from the transformation of melanin cells, is characterized by the highest burden of mutations (98). Current clinical treatment for melanoma primarily involves local resection surgery followed by radiotherapy to eliminate residual cancer cells. In 2005, Brinkman et al. utilized the major structural protein VP1 of polyomavirus to construct recombinant VLPs carrying epitopes of ovalbumin (OVA257-264) or autoantigen tyrosinase-associated protein (TRP2180-188) through gene fusion. These VLPs vaccines were successfully employed in a murine melanoma model and demonstrated their ability to induce CTL responses (99).

Gp100 is a melanoma-associated antigen involved in mature melanin synthesis (100). Katrin Kramer utilized rabbit hemorrhagic virus (RHDV) VLPs to prepare vaccine particles with varying Gp100 copy numbers: gp100.1L, gp100.2L, and gp100.3L. All three vaccine formulations successfully induced the proliferation of CD8^+^ T cells, with gp100.2L and gp100.3L significantly enhancing IFN-γ production. Mice vaccinated with either gp100.2L or gp100.3L exhibited effective and specific anti-tumor immune responses. The RHDV-VLPs employed in this study are composed of 180 copies of the viral capsid protein VP60 and can be recombined to express repeated tumor epitopes. Importantly, the parental rabbit hemorrhagic virus is not derived from humans, thereby circumventing the issue of pre-existing immunity in human subjects and positioning it as a potential antigen delivery platform (79). In another related clinical study, the MelQbG10 vaccine was evaluated in a phase I/II trial involving patients with stage II/IV melanoma, incorporating various adjuvants. Results indicated that most patients, regardless of disease stage, elicited T cell-specific responses, with enhanced responses observed in conjunction with adjuvant therapy. However, some patients experienced a loss of Melan-A antigen expression in tumor cells, underscoring the necessity for multiple antigen peptides to effectively suppress or eliminate tumor growth (101). To address this challenge, Keman Cheng’s team employed genetic engineering techniques to incorporate gp100 (KVPRNQDWL) or the model antigen OVA257-264 (SIINFEKL) into the HBV core protein, resulting in the development of a dual antigen delivery system based on HBcAg-VLPs (80). This dual antigen delivery system for cancer immunotherapy introduces a novel concept by simultaneously presenting different types of antigens, which may enhance the efficacy of overcoming immune tolerance in tumor cells.

### 5.3 Cervical cancer

HPV, particularly HPV16 and HPV18, is the primary etiological factor in cervical cancer, accounting for 70% of cases (102). Initially, VLPs were mainly used as a prophylactic vaccine to prevent cervical cancer caused by HPV infection (103, 104). However, VLPs-based cancer vaccines are still in the pre-clinical stage. As early as 1998, H L Greenstone et al. developed VLPs carrying the E7 protein of HPV by incorporating it into the major capsid protein L1 along with minor capsid protein L2 or fusion proteins such as E7-E2 (43 kDa). In a prevention experimental model, C57BL/6 mice pretreated with VLPs were protected from challenge with TC-1 tumor cell line expressing HPV-E7 (105). Alberto Monroy-Garcia has successfully produced VLPs fused with E6 or E7 T cell epitopes in plant cells, and the results have also demonstrated their therapeutic potential in tumor models (83).

## 6 Conclusion

Cancer immunotherapy has recently witnessed significant advancements, with VLPs emerging as a promising platform for cancer treatment. VLPs possess unique advantages that make them prominent in cancer vaccine research. By mimicking viral capsids, VLPs can carry and display tumor-associated antigens, making them potent immune activators. Unlike traditional cancer vaccines, VLPs have the unique capability to elicit both humoral (B cell) and cellular (T cell) immune responses. This dual activation broadens the therapeutic potential of VLPs in cancer treatment, particularly by stimulating the immune system to generate long-lasting immune memory.

In terms of clinical application, research on VLPs is transitioning from conventional prophylactic vaccines toward therapeutic vaccine development. The objective of therapeutic VLPs-based vaccines is to stimulate the patient’s immune system to recognize and eliminate tumor cells expressing specific tumor-associated antigens. However, therapeutic vaccines face significant challenges in inducing an effective immune response in individuals with established tumors, due to mechanisms employed by tumors to evade immunity. Ongoing research focuses on the precise design of VLP structures to enhance their capability to present tumor antigens and improve overall immunological efficacy. Recent studies highlight the combination of VLPs with other anticancer agents, such as immune-stimulating agents, antibodies, or immune checkpoint inhibitors, to augment therapeutic efficacy. This strategy not only intensifies immune responses but also improves the durability of treatment by overcoming tumor immune escape mechanisms. Despite the immense potential of VLPs in cancer immunotherapy, several challenges remain. Cancer cells often evade immune system detection by expressing immune-suppressive molecules, such as PD-L1, which may limit the therapeutic effectiveness. It is crucial to develop novel strategies to overcome immune escape mechanisms, including combining VLPs with immune checkpoint inhibitors. Furthermore, the tumor microenvironment and antigen expression vary greatly among patients, posing a substantial challenge in developing a universal VLPs-based vaccine. Future research may prioritize developing personalized VLPs-based vaccines tailored to individual characteristics, including tumor neo-antigen profiles and immune cell infiltration, to enhance treatment effectiveness. Additionally, the production of VLPs necessitates intricate cell culture and protein expression systems, resulting in relatively high costs. Enhancing production efficiency and reducing expenses are pivotal for clinical application. As a cancer immunotherapy platform, VLPs are rapidly advancing and exhibiting promise across various cancer types. Despite challenges like immune evasion, tolerance, and production costs, these issues expected to be mitigated through ongoing technological advancements. Combining VLPs with immune checkpoint inhibitors and other immunotherapies may emerge as a key direction for future cancer treatments. Personalized treatment strategies can further enhance the role of VLPs in cancer therapy by promoting long-term immune memory and addressing drug-resistant tumors.

In conclusion, despite being in the early stages of research and clinical trials, VLPs offer a promising avenue for cancer immunotherapy with an extensive range of potential applications. Hence, further exploration and attention from the scientific and medical communities are warranted.
